# Functional family therapy across the COVID-19 pandemic

**DOI:** 10.3389/fpsyg.2025.1531738

**Published:** 2025-06-02

**Authors:** Tor Løstegaard Hagen, Tony C. A. Tan, Dagfinn Mørkrid Thøgersen, Gunnar Bjørnebekk

**Affiliations:** ^1^Department of Special Needs Education, University of Oslo, Oslo, Norway; ^2^Centre for Educational Measurement, University of Oslo, Oslo, Norway; ^3^Norwegian Center for Child Behavioral Development (NUBU), Oslo, Norway; ^4^The Norwegian Institute for Public Health, Oslo, Norway

**Keywords:** conduct problems, adolescents, family therapy, functional family therapy, treatment outcome, COVID-19

## Abstract

The present study examines the effectiveness of the Functional Family Therapy (FFT) program in Norway through the COVID-19 pandemic using archival data. Since Norway's national lockdowns imposed significant disruptions on program delivery, concerns arose regarding FFT's effectiveness during the public health emergency; however, this hypothesis remained untested. This exploratory study uses a multigroup quasi-experimental design by comparing clients before and during the COVID-19 pandemic. The data includes 518 adolescents and their families referred to FFT for serious and persistent antisocial behavior. The mean age of the youth was 14.2 years and the representation between boys and girls was close to equal (49.4% girls). Statistical analyses showed that clients' attributes at admission remained stable, and FFT remained effective in bringing about behavioral improvements and risk reductions throughout the pandemic. Additionally, regression results suggested that older clients tended to have greater reductions in behavioral problems and risk levels before lockdowns; however, this age effect disappeared after the onset of COVID. Clients returning home from institutional care reported stronger behavioral gains, whereas those living in foster homes showed less favorable risk outcomes. Collectively, these results suggest that both the FFT target population and treatment outcomes remained stable despite disruptions in program delivery during the pandemic.

## Introduction

Behavioral problems among young Norwegians represent significant costs to both individuals and society. Between 3 and 5 percent of youths in Norway are reported to have serious behavioral challenges (Berg et al., 2020). The literature generally categorizes behavioral pathology into internalizing and externalizing manifestations, with the latter being characterized by aggressive behavior, drug use, school absenteeism, or relationship problems within families (Hartnett et al., 2017). A high level of norm-violating behavior in adolescence is predictive of criminal involvement later in life (Lanctôt et al., 2007), substance abuse, and depressive symptoms (Wiesner et al., 2005). It is therefore of great importance to provide effective interventions to adolescents with these types of difficulties. Systemic family therapy appears to be effective in this context (Carr, 2020; Dopp et al., 2017), and one of the therapies often recommended is Functional Family Therapy (Robbins and Alexander, 2021).

On March 12, 2020, the Norwegian Directorate of Health declared a nationwide lockdown in response to the escalating COVID-19 crisis. This decision had a major impact on all institutions, permitting only critical social infrastructure to operate. Unfortunately, child and family welfare services such as Functional Family Therapy (FFT) were not included in the list of authorized operations. As a result, all interventions were suspended for the first 2 weeks and severely restricted for several months thereafter (Øverli and Gundersen, 2020). Although new regulations from the Directorate weeks later allowed the reactivation of physical sessions with appropriate precautions in place, therapists were unable to treat clients as usual, creating significant barriers to professional services (Taraldsen, 2020). The COVID-19-induced emergency measures in Norway were not fully lifted until late September 2021, resulting in 18 months of FFT delivered in altered forms.

Such gaps in services raise doubts about whether therapists succeeded in engaging and creating a working alliance with family members, especially during the crucial engagement phase of treatment. In a qualitative study conducted in the UK, FFT professionals reported challenges in building engagement and forming a therapeutic alliance in the absence of physical meetings (Lange et al., 2023). Firstly, therapists were deprived of opportunities to observe family interactions. Secondly, therapist-client interactions over computer screens served as a poor substitute for natural conversations, let alone involving other family members in the process. Thirdly, the additional technical and administrative demands on therapists could hinder their ability to engage with and respond appropriately to clients and their families. A study evaluating a similar family welfare program during COVID-19 in the USA reported reduced activity levels and lower completion rates, highlighting the challenges of maintaining client engagement without physical contact (Rybińska et al., 2022).

Despite all its profound negative impact, the COVID-19 pandemic presented a unique research opportunity. It served as a natural experiment for testing FFT's effectiveness by introducing significant variations in treatment conditions. It also provided researchers and policymakers an opportunity to stress test the implementation of FFT in Norway during turbulent times. Lockdowns and restrictions severely altered people's routines, a known risk factor for vulnerable individuals receiving FFT, as well as potentially disrupting the quality of service provided by therapist. A more in-depth understanding of youths' and families' experiences during this health emergency could contribute to the knowledge and enhancement of FFT's effectiveness and implementation robustness.

The aim of this study is to explore how FFT services in Norway were affected by the COVID-19 pandemic. The study will first enquire whether clients differed at admission before vs. after the onset of COVID-19; then it will ask whether clients' outcomes have changed at discharge during the COVID-19 pandemic. To address the question of whether the intake for treatment changed after the outbreak of COVID, we test for group differences in risk, problem burden, age, gender, proportion of immigrants, previous interventions/treatments received, and whether the adolescents have been in foster care or institutions, by comparing youth that started FFT before the outbreak with those starting after. Next, this study will investigate variables associated with treatment-phase outcomes and long-term maintenance of outcomes and compare these results for youths receiving FFT prior to pandemic, with those receiving FFT during. This article will also reflect on methodological strengths and weaknesses, as well as policy implications and directions for future research.

### Functional Family Therapy (FFT)

Functional Family Therapy (FFT) was established in Norway in 2007 as a supplemental and complementary service to Multisystemic Therapy (MST)—a more intensive and resource-demanding intervention that has been in operation since 1999—by offering interventions to clients with less severe behavioral issues (Hukkelberg et al., 2022). The objective of FFT is to reduce the development of youths' challenging and antisocial behaviors such as violence, family conflict, school absenteeism, substance abuse, and criminal offenses (Thøgersen et al., 2022). FFT is an evidence-based, certified Blueprint intervention program for youth with, or at risk of developing, conduct problems (Mihalic et al., 2004).

FFT is grounded in several theoretical frameworks including systemic theory, behavioral theory, and cognitive theory. FFT posits that problematic behavior in youth is a symptom of family dysfunctions. By addressing the root causes in the family, therapists can help family members improve their individual functioning and strengthen their relationships. Techniques such as developing more effective, positive communication strategies and problem-solving skills are frequently modeled in FFT (Alexander et al., 2013).

Meta-analyses support the effectiveness of FFT by showing significant behavioral improvements in the treated group compared to the control group (Baldwin et al., 2012; Hartnett et al., 2017). Additionally, FFT appears to provide protection beyond the referred youth by demonstrating risk reduction for siblings, inoculating them from repeating maladaptive behavior (Robbins and Midouhas, 2021). Furthermore, FFT has been shown to outperform other established interventions (Baglivio et al., 2014; Baldwin et al., 2012) with long-lasting effects (Hukkelberg et al., 2022). In contrast to these highly encouraging results related to the effects of FFT, recent findings from an randomized controlled trial (RCT) conducted in Child Welfare Services in Norway (Olseth et al., 2024), two meta-analyses examining FFT's effects on broader behavioral issues (Hunkin et al., 2024; Littell et al., 2023), and a scoping review of severe and persistent conduct problems (Lee et al., 2024) demonstrated only possible clinically important effects for some outcomes.

The FFT treatment model stresses the importance of establishing and maintaining good relationships between therapists and family members early in treatment (Alexander et al., 2013). Despite high dropout rates among clients with behavioral difficulties, FFT has demonstrated an ability to retain ~83% of families through to completion (Baglivio et al., 2014; Sexton and Alexander, 2004; White et al., 2013). Potential moderators of treatment effect have been found in studies demonstrating better outcomes in families that were open-minded and motivated to receive help (McPherson, 2017), and when therapists adhered to FFT protocols (Robbins et al., 2016; Sexton and Turner, 2011).

Another key feature of the FFT design is its quality assurance (AQ) routines that is implemented as a part of training therapists and supervisors in the practice of the intervention. This relies on continuous data collection through treatment planning and evaluation from the therapist, systematic feedback on treatment progress from family members and weekly group supervision on therapist practice. The QA system aims to identify and rectify challenges related to treatment motivation, targeted behavior changes and sustainment of family change at the earliest stage.

### The current study

This exploratory study aims to investigate the reach and effectiveness of FFT during the COVID-19 pandemic, addressing the following research questions:

**Research Question 1:** Did youths' characteristics change at admission during COVID-19?
**Research Question 2:** Was FFT treatment effective in improving behavioral outcomes and reducing reoffending risks during the COVID-19 pandemic?
**Research Question 3:** Which demographic variables, living arrangements, and/or mental health conditions were associated with FFT outcome measures at discharge?
**Research Question 4:** During COVID-19, which variables were associated with youths' long-term behavioral improvement?

## Methods

### Participants

For the purposes of this study, we studied anonymized clinical records of clients who entered FFT in Norway between March 13, 2018, and August 31, 2021, totaling 553 adolescents. The data was retrieved from the quality assurance database for FFT in Norway operated by Norwegian Center for Child Behavioral Development (NCCBD). Thirty-five cases were excluded from further analyses due to missing data at either admission or discharge, representing a loss rate of 6.33%. The final sample size was 518, with balanced representation between sexes (49.4% girls) and a mean age of 14.2 years.

To be offered FFT in Norway a youth between the ages of 11 and 19 must be referred to an FFT team. Tour FFT teams are in the Child Welfare Service (CWS) sites in the southern, eastern, and central parts of Norway. The Norwegian CWS is organized as a two-tiered system, comprising both municipal and regional levels. Three FFT teams operates at the regional level, with referrals coming from municipal CWS. The fourth team is a municipal service, where referrals come from several different sources, including parents seeking help, schools, the police, and social services. A fifth FFT team is organized in the Family Counseling Services (FCS) also allows for family self-referral to FFT. The inclusion criteria for FFT are: (a) high family conflict with parental complaints about challenging youth behavior, (b) moderate to severe behavior problems (e.g., truancy, verbal aggression, violence, criminal behavior) or (c) dysfunctional behavior at school characterized by prolonged absenteeism, sustained conflicts with peers, profound psychological challenges, or the initiation of drug abuse. The FFT exclusion criteria are: (a) living alone without primary caregivers, (b) suicidal, psychotic, or at imminent risk of self-harm or harm to others, or (c) intellectual disability as the sole reason for referral.

### Procedures

The FFT therapist conducts an independent evaluation of each referred client's status through clinical consultations at admission (initial time point, *T*_0_), with any disagreements against referral information reconciled together with the FFT team leader. This multi-informant co-assessment design promotes measurement accuracy. All FFT therapists are qualified practitioners with additional training specific to this program and must follow detailed guidelines when conducting the assessment. At discharge (*T*_1_), the FFT therapist makes a concluding assessment of clients' behavioral outcomes and risk of reoffending, following the same guidelines used at admission. The FFT team leaders are responsible for reporting the pre- and post-data, as well as other treatment data to the Primula database at NUBU. After treatment, an independent quality assurance unit interviews parents on the clients' progress through three phone interviews, thereby collecting data on the maintenance of behavioral outcomes at 6-, 12-, and 18-months post-discharge (*T*_2_-*T*_4_).

### Measures

#### Outcome variables

Treatment effectiveness is an abstract concept. Any action resulting in an improvement in clients' social functionality or wellbeing can serve as a basis for measuring a program's efficacy. The Norwegian FFT team followed international practices by establishing a dual-indicator system consisting of clients' non-engagement with serious behavior problems (e.g., drug use, criminal behavior and violence) and reoffending risk scores. If both indices are valid measures of the latent construct of “FFT effectiveness,” the two manifest variables would covary strongly across individuals and over time (Lord and Novick, 1968).

FFT's effectiveness can be quantified using two measures: increases in the number of FFT's ultimate goals (non-engagement with serious behavior problems, BHV) and reductions in reoffending risks (RSK). BHV is operationalized as the sum score of the following five binary items: whether the youth (a) lives with their parents or legal guardians: is not incarcerated, or has been placed in foster care or another accommodation arranged by the CWS; (b) goes to school or work: is attending school (not truant) or vocational training, or is in combined school/training in a company, or is of compulsory school age and has a job (at least half-time); (c) complies with the law: has not been arrested, institutionalized, or incarcerated, and will not be appearing in a conflict resolution board for offenses or violations (other forms of social reactions may include being apprehended by a security guard, reporting to the police or child welfare services, or concern discussions related to clearly demonstrated offenses committed by the young person); (d) abstains from drug use: does not use any substances in a way that impairs daily functioning or lead to other serious consequences—at home, at school, or in relation to friends or the local community; and (e) refrains from violence: does not use violence or threats of violence (NUBU, 2021). Combining the items lead to a possible range between 0 and 5. Each client's BHV was measured at admission (*T*_0_), discharge (*T*_1_), as well as at follow-ups (*T*_2_ to *T*_4_). For the purpose of testing FFT effectiveness, the change in BHV was defined as the difference score between discharge and admission (ΔBHV = BHV1 – BHV0).

The FFT teams employed Hoge and Andrews's (2011) Youth Level of Service (YLS) to measure clients' reoffending risks. Using 42 binary items, this inventory scores youths' criminogenic risk along the following subscales: (a) previous or current law offenses, (b) family relations, (c) education/work, (d) friendship, (e) substance abuse, (f) leisure/recreation, (g) personality/behavior, and (h) attitudes/orientation. Since YLS sum scores are positively correlated with risks of reoffending, clients tended to report higher scores at admission than at discharge, with the change score defined as: ΔRSK = RSK0 – RSK1. Unlike behavioral indicators, the FFT team only documented risk measures at admission and discharge, not at follow-ups.

#### Covariates

In addition to any treatment effect, clients' responses to interventions may vary due to individual differences such as demographic attributes, living arrangements, and mental conditions. This study has therefore controlled for such variations in background variables with the aim of isolating the effects solely attributable to the FFT interventions. Robbins et al. (2016) reported that the effectiveness of FFT varied systematically depending on clients' sex and age. Although FFT has been shown to be effective across cultures (Gan et al., 2021), a recent OECD report highlighted the differential impact of COVID-19 on persons with immigration backgrounds (OECD, 2022). Consequently, the variables AGE, FEMALE, IMMI1, and IMMI2 were included in all analyses. The first-generation immigrants (IMMI1) variable refers to individuals born outside of Norway, while the second-generation immigrants (IMMI2) variable refers to those born in Norway to parents who were both born broad. Treatment effectiveness can also be sensitive to clients' living arrangements. A study commissioned by the Norwegian Directorate for Children found that youths in institutional care are more likely to commit crimes than those in foster homes (Drange et al., 2022). It is therefore important to identify clients from foster care (FOSTER) or institutional care (INSTI) in regression analyses. The variable FOSTER refers to whether the young person is living in foster care during treatment, and the INSTI variable refers to whether FFT has been initiated in the process of transitioning back to the home after a stay in a child welfare institution.

Lastly, clients' mental conditions should also be taken into consideration. This study recorded whether it was the client's first time receiving FFT or if they had previously undergone FFT or similar interventions (PRIOR). Since Norwegian youths experienced significant psychological hardship during COVID-19 (Nøkleby et al., 2023), we also controlled for youth currently or previously receiving mental health services (PSYC). Supplementary Table B1 summarizes all variables used in this study.

### Group division

Participants were divided into three groups relative to Norway's national lockdown on 12 March 2020. Clients in Group A entered the FFT program between 13 March 2018 and 12 March 2019; COVID-19 had no impact on members of this group during either the treatment or maintenance phase. Group B started FFT between 13 March 2019 and 12 March 2020; although COVID-19 did not impact this group's treatment, it interfered with its maintenance period. Lastly, youths who entered FFT between 13 March 2020 and 31 August 2021 were labeled Group C; members of this group experienced disruptions in FFT delivery during both the treatment and maintenance phases.

### Analytic procedures

Any missing data were first treated using Mplus's default multiple imputation procedures (van Buren, 2018), where ten versions of the imputed datasets were analyzed separately, then pooled together using Rubin's Rules (Rubin, 1987) for accurate point estimates and standard errors. Next, all models employed the robust maximum likelihood estimator (MLR; Muthén and Muthén, 2017) due to its ability to withstand heteroscedasticity and non-normal residuals.

Group differences at admission were examined using one-way analysis of variance (ANOVA) (R Core Team, 2023). R was also used for investigating FFT effectiveness through data visualization and frequency tables.

#### Models

This study examined both the short-term and long-term effectiveness of FFT during the COVID-19 pandemic using the following three models:

I. *A Linear Model using Behavioral Improvement as an Effectiveness Measure*.
  This regression investigated the immediate impact of FFT at discharge relative to admission using changes in behavioral indicators as the outcome variable. Clients' individual attributes were also controlled as covariates using the following model equation:
  (1) $$\begin{array}{l} \Delta \text{BHV}=\beta_{0}+{\text{β}}_{1}\text{AGE}+\beta_{2}\text{FEMALE}+\beta_{3}\text{IMMI}1\\ \;+{\text{β}}_{4}\text{IMMI}2+\beta_{5}\text{PRIOR}+\beta_{6}\text{INSTI}\\ \;+{\text{β}}_{7}\text{FOSTER}+{\text{β}}_{8}\text{PSYC}+\epsilon \end{array}$$
II. *A Linear Model using Risk Reduction as Effectiveness Measure*
  Model 2 differed from Model 1 by the outcome variable, where reductions in reoffending risks replaced behavioral changes as the effectiveness indicator:
  (2) $$\begin{array}{l} \Delta \text{RSK}=\beta_{0}+{\text{β}}_{1}\text{AGE}+\beta_{2}\text{FEMALE}+\beta_{3}\text{IMMI}1\\ \;+{\text{β}}_{4}\text{IMMI}2+\beta_{5}\text{PRIOR}+\beta_{6}\text{INSTI}\\ \;+{\text{β}}_{7}\text{FOSTER}+{\text{β}}_{8}\text{PSYC}+\epsilon \end{array}$$
III. *A Latent Growth Curve Model for Measuring Long-term Effectiveness*

**Figure 1:**
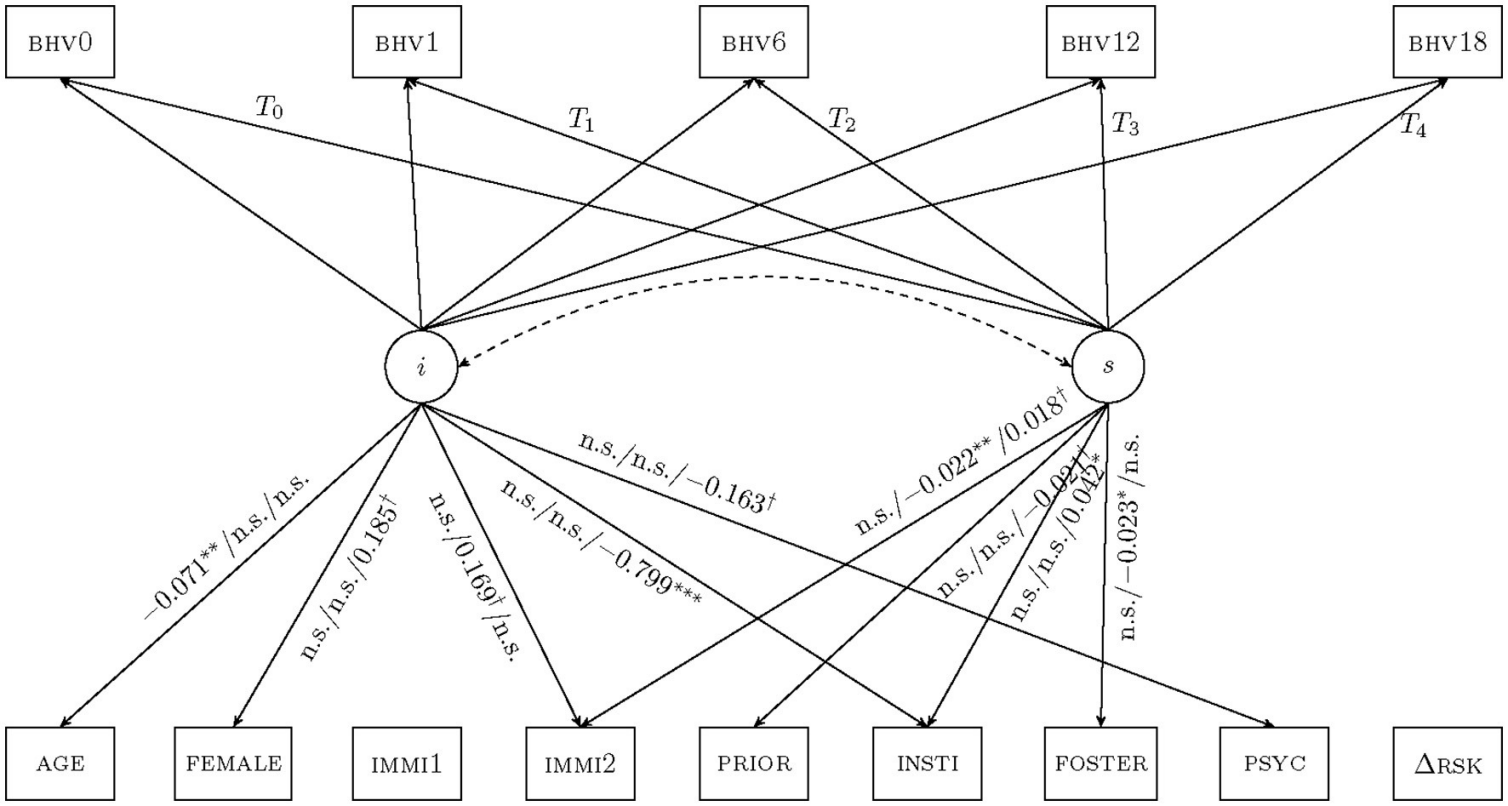
Latent growth curve models for FFT effectiveness. Note: These latent growth curve models evaluated variables associated with FFT effectiveness through the COVID-19 pandemic. The intercept (*i*) carries factor loadings of 1s (omitted) to all outcome variables while the slope (*s*) carries time stamps measured in months as factor loadings from *T*_0_ to *T*_4_. Intervals between admission (*T*_0_) and discharge (*T*_1_) vary by clients. All subsequent time points are 6 months apart, representing 6-, 12-, and 18-months follow-up measures. The dashed arrow suggests no significant covariations between intercepts and slopes, hence growth curves do not fan in or out. Unstandardized coefficients are reported in the order Group *A*/*B*/*C*. Covariates receiving no arrows provide insufficient explanatory power for growth curves. n.s., not significant at α = 0.10 level, †*p* < 0.10, *p < 0.05, **p < 0.01, ***p < 0.001.

Latent growth curve models (LGM; Bollen and Curran, 2006) are particularly suitable for examining FFT's longitudinal effect on clients' behavior. This study included five time-point measures of BHV (*T*_0_ to *T*_4_) as the LGM outcome variables, as well as covariates similar to the regression models. In addition, clients' changes in risks (ΔRSK) were also included as the final covariate in the LGM to partial out the correlations between the two effectiveness indicators (Hukkelberg et al., 2022). The LGM intercepts were assigned factor loadings of 1, while the slopes received factor loadings from *T*_0_ to *T*_4_. Covariates' factor loadings were freely estimated, and their unstandardized parameters are reported in Figure 1.

## Results

### Descriptive statistics

Table 1 revealed that not all 518 clients fully participated at every time point, with attrition rates of 27.4%, 13.3%, and 15.0% for the 6-, 12-, and 18-month follow-ups, respectively. Secondly, behavioral measures appeared to have experienced a ceiling effect, with clients entering FFT with an average BHV0 = 4.1 on a scale with a maximum of 5 (see Supplementary Table B1). The mean behavioral indicator increased at discharge to BHV1 = 4.8 and remained stable during the maintenance phase. All three groups showed significant reductions in the risk indicator at discharge relative to admission. Lastly, both the means and standard deviations of the effectiveness indicators, ΔBHV and ΔRSK, trended downwards, suggesting possible intragroup convergences over time.

**Table 1:** Descriptive statistics.

**Variable**	***N***				**M**				**Mdn**				**SD**			
BHV0	**518**	147	159	212	**4.1**	4.0	4.2	4.1	**4**	4	4	4	**1.0**	1.0	0.9	0.9
BHV1	**518**	147	159	212	**4.8**	4.8	4.8	4.7	**5**	5	5	5	**0.6**	0.6	0.4	0.6
BHV6	**376**	107	111	158	**4.7**	4.7	4.7	4.6	**5**	5	5	5	**0.7**	0.7	0.5	0.8
BHV12	**326**	96	107	123	**4.7**	4.8	4.8	4.6	**5**	5	5	5	**0.7**	0.4	0.4	0.8
BHV18	**277**	90	110	77	**4.7**	4.8	4.7	4.6	**5**	5	5	5	**0.7**	0.5	0.6	0.8
RSK0	**518**	147	159	212	**11.7**	11.8	11.1	12.1	**12**	11	11	12	**5.4**	5.5	5.2	5.3
RSK1	**518**	417	159	212	**6.0**	5.9	5.1	6.7	**5**	5	4	6	**4.5**	4.6	3.9	4.5
AGE	**518**	147	159	212	**14.2**	14.0	14.5	14.1	**15**	14	15	14	**1.9**	2.0	1.8	1.8
FEMALE	**518**	147	159	212	**0.5**	0.5	0.5	0.5	**0**	0	0	1	**0.5**	0.4	0.5	0.5
IMMI1	**518**	147	159	212	**0.1**	0.1	0.1	0.1	**0**	0	0	0	**0.3**	0.3	0.3	0.3
IMMI2	**518**	147	159	212	**0.1**	0.1	0.2	0.1	**0**	0	0	0	**0.3**	0.2	0.3	0.3
PRIOR	**518**	147	159	212	**0.1**	0.1	0.1	0.1	**0**	0	0	0	**0.3**	0.3	0.3	0.3
INSTI	**518**	147	159	212	**0.1**	0.1	0.1	0.1	**0**	0	0	0	**0.3**	0.3	0.3	0.3
FOSTER	**518**	147	159	212	**0.1**	0.1	0.1	0.1	**0**	0	0	0	**0.3**	0.2	0.2	0.2
PSYC	**518**	147	159	212	**0.6**	0.6	0.6	0.6	**1**	1	1	1	**0.5**	0.4	0.4	0.4
ΔBHV	**518**	147	159	212	**0.7**	0.7	0.7	0.6	**0**	1	0	0	**0.9**	1.0	1.0	0.9
ΔRSK	**518**	147	159	212	**5.7**	5.9	5.9	5.4	**5**	5	5	5	**4.1**	4.8	4.0	3.5
**Variable**	**Skewness**				**Excess kurtosis**				**Minimum**				**Maximum**			
BHV0	**−1.1**	−1.2	−1.0	−1.0	**1.0**	1.6	0.2	0.7	**0**	0	1	1	**5**	5	5	5
BHV1	**−3.2**	−3.4	−4.3	−2.6	**11.7**	13.3	24.7	6.6	**1**	1	1	2	**5**	5	5	5
BHV6	**−2.9**	−4.2	−2.4	−2.3	**10.5**	24.2	6.3	5.3	**0**	0	2	1	**5**	5	5	5
BHV12	**−3.1**	−3.0	−2.5	−2.6	**12.7**	10.9	5.3	8.0	**0**	2	3	0	**5**	5	5	5
BHV18	**−2.9**	−2.6	−2.2	−3.1	**11.0**	6.6	4.6	11.9	**0**	2	2	0	**5**	5	5	5
RSK0	**0.4**	0.6	0.4	0.2	**0.2**	0.8	0.1	−0.1	**0**	0	1	1	**29**	29	27	29
RSK1	**1.2**	1.3	1.6	0.9	**1.9**	2.0	4.1	1.0	**0**	0	0	0	**26**	24	26	25
AGE	**−** **0.7**	−0.9	−0.9	−0.3	**0.7**	0.9	1.9	−0.5	**6**	7	6	9	**18**	18	18	18
FEMALE	**0.0**	0.2	0.0	−0.1	**−2.0**	−2.0	−2.0	−2.0	**0**	0	0	0	**1**	1	1	1
IMMI1	**2.5**	2.6	2.4	2.4	**4.1**	4.9	4.0	3.6	**0**	0	0	0	**1**	1	1	1
IMMI2	**2.3**	2.9	1.8	2.5	**3.5**	6.4	1.3	4.3	**0**	0	0	0	**1**	1	1	1
PRIOR	**2.5**	2.5	2.4	2.4	**4.1**	4.3	4.0	4.0	**0**	0	0	0	**1**	1	1	1
INSTI	**2.3**	2.5	2.1	2.3	**3.2**	4.3	2.4	3.3	**0**	0	0	0	**1**	1	1	1
FOSTER	**3.3**	3.7	3.2	3.2	**9.1**	11.4	8.3	8.3	**0**	0	0	0	**1**	1	1	1
PSYC	**−** **0.4**	−0.3	−0.3	−0.5	**−** **1.9**	−1.9	−1.9	−1.7	**0**	0	0	0	**1**	1	1	1
ΔBHV	**0.9**	1.0	0.6	0.9	**1.9**	2.2	1.4	1.4	**−3**	−2	−3	−2	**5**	5	4	4
ΔRSK	**0.6**	0.9	0.1	0.3	**1.5**	1.5	1.8	−0.3	**−12**	−7	−12	−2	**25**	25	19	16

This table summarizes the descriptive statistics for all variables used in this study. Statistics are presented in the order **overall**/A/B/C relative to Norway's national COVID−19 lockdown.

Across the three groups, clients had mean ages slightly above 14 years, with balanced representation between the two sexes. Approximately 10% of the participants were born outside of Norway, and another 10% were born to parents who were both born overseas. Similar percentages were reported for clients living in foster care and former institutional care facilities. Although 10% of the youths had previously undergone FFT or similar treatment, a significantly higher proportion had received mental health services, at approximately 60%.

The three groups in this study (A: fully prior, B: partly prior and C: during the pandemic) were rather similar at admission. One-way ANOVA in Table 2 showed stable client profiles before and during COVID-19, with marginal differences in age (*F*_2, 515_ = 2.7, *p* = 0.07).

**Table 2:** Analysis of variance table.

**Variable**	**ν*_1_***	**ν*_2_***	***F*(ν_1_, ν_2_)**	***p*–value**
BHV0	2	515	0.654	0.520
BHV1				
BHV6				
BHV12				
BHV18				
RSK0	2	515	1.727	0.179
RSK1				
AGE	2	515	2.669	0.070
FEMALE	2	515	0.554	0.575
IMMI1	2	515	0.111	0.895
IMMI2	2	515	2.265	0.105
PRIOR	2	515	0.010	0.990
INSTI	2	515	0.307	0.736
FOSTER	2	515	0.160	0.852
PSYC	2	515	0.763	0.467
ΔBHV				
ΔRSK				

Group differences at intake for selected variables are presented in the one–way ANOVA table, with ν_1_ and ν_2_ representing the numerator and denominator–degree of freedoms respectively.

^†^*p* < 0.10.

The correlation of the variables in the study, Table 3 shows that the overall behavioral indicator BHV score had strong intertemporal correlations across different measurement points, with correlation coefficients (*r*) ranging from 0.27 to 0.71. These correlations appeared to have strengthened as clients entered the maintenance phase, suggesting stable and enduring recoveries (*r* range from 0.54 to 0.71). The risk indicator RSK also correlated strongly with itself at admission and discharge (*r* = 0.66). Due to its reverse scale, RSK negatively correlated with BHV (with *r* values ranging from −0.24 to −0.56), supporting construct validity through close and stable associations between these two variables in indicating FFT effectiveness (Shadish et al., 2002). Thirdly, behavioral improvement (ΔBHV) was highly but negatively correlated with initial behavior (BHV0, *r* = −0.81); the fact that only those starting treatment with low BHV scores showed significant improvement suggested a possible ceiling effect in the behavior scale. Lastly, weak correlations between covariates suggested low risks of multicollinearity (Wooldridge, 2020).

**Table 3:**
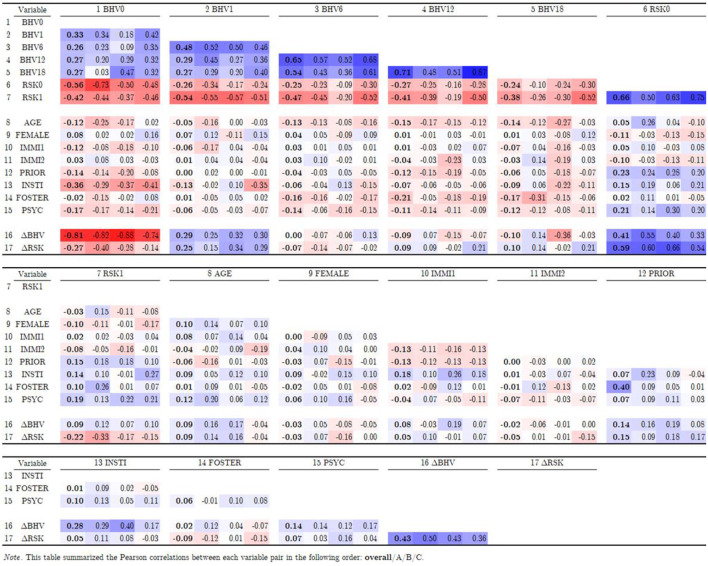
Correlation table.

### FFT effectiveness measures

**Figure 2:**
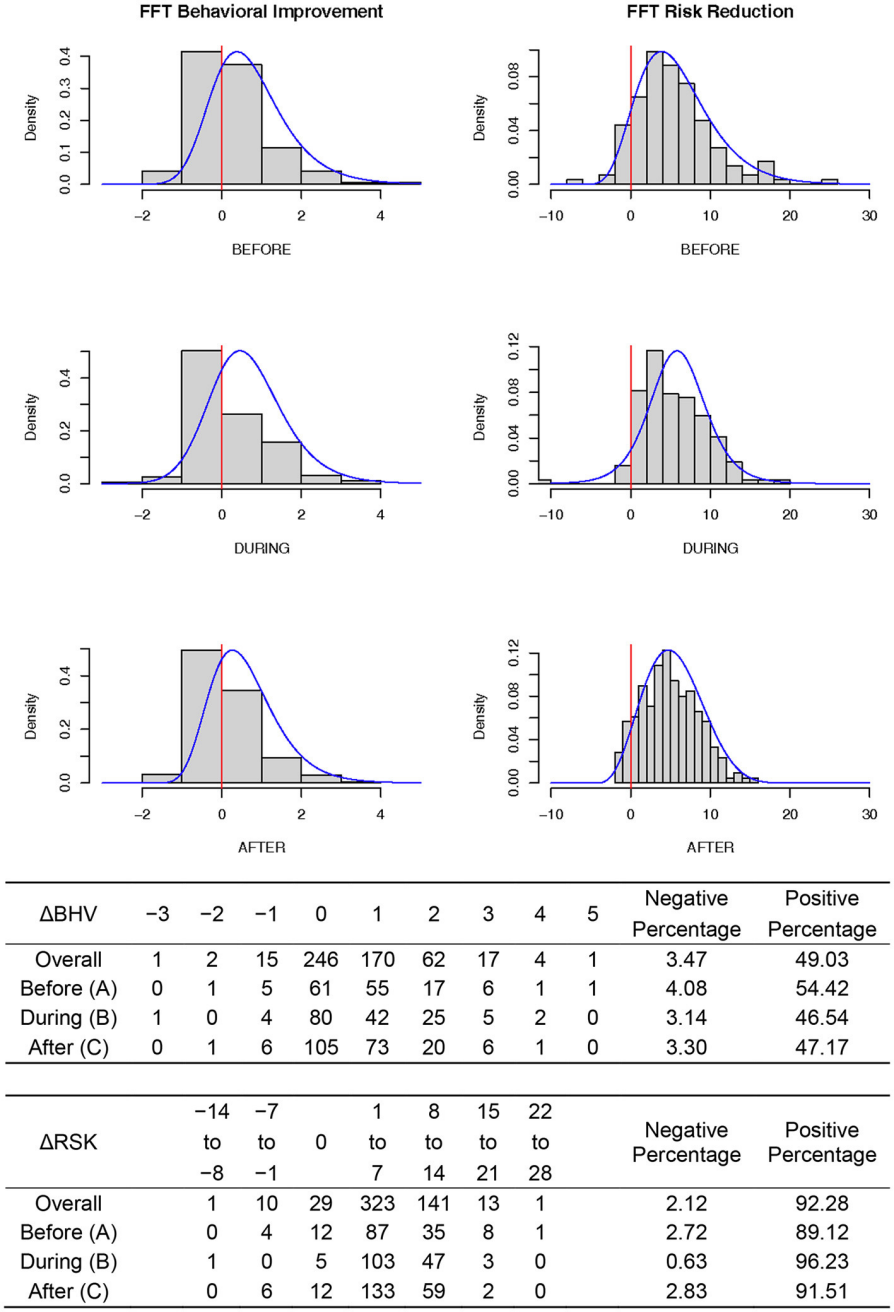
Distributions of FFT effectiveness measures. Note: These distribution plots summarized the frequencies of behavioral improvement (ΔBHV) and risk reductions (ΔRSK), two indicators of FFT effectiveness. The same information was produced in tabular form to facilitate percentage comparisons.

The study operationalized treatment effectiveness as the dual indicators of behavioral improvement and risk reduction. Figure 2 illustrates the frequency distributions of these two measures by indicators (columns: left = ΔBHV; right = ΔRSK) and by groups (rows: top = Group A; middle = Group B; bottom = Group C). The histograms show improvements for most of the clients, with close to 95% reporting reductions in reoffending risks, a key goal of FFT treatment. Possibly due to the ceiling effect, the ΔBHV scale was more difficult to interpret, with just under 50% reporting positive changes in behavioral sum scores and another 45% reporting no changes. This result is likely to be a result of the initial high scores on this scale (*M* = 4.1) and suggest that this scale might not be well suited to measure the challenges families face when being referred to FFT in Norway.

### Regression models

An *R*^2^ statistic reports the proportion of variations in the outcome variable that can be explained by the variations of the input variables. Higher *R*^2^ generally indicates stronger explanatory power among the predictive variables. Multigroup comparisons in Table 4 revealed elevated *R*^2^ in both effectiveness indicators for Group B (${\text{R}}_{\Delta \text{BHV}}^{2}$ = 0.238 and ${\text{R}}_{\Delta \text{RSK}}^{2}$ = 0.110) compared to those for Group A (${\text{R}}_{\Delta \text{BHV}}^{2}$ = 0.135 and ${\text{R}}_{\Delta \text{RSK}}^{2}$ = 0.076) and Group C (${\text{R}}_{\Delta \text{BHV}}^{2}$ = 0.081 and ${\text{R}}_{\Delta \text{RSK}}^{2}$ = 0.090), suggesting COVID-19 has introduced more uncertainty into the models. Additionally, intercepts for both models turned significant after the pandemic onset ($\beta_{0}^{\text{C}}$ = 0.977, *p* < 0.10 for ΔBHV and $\beta_{0}^{\text{C}}$ = 2.040, *p* < 0.001 for ΔRSK). Such upward shifts in regression lines suggested structural improvement in youth's behavioral outcomes in addition to any demographic variables.

**Table 4:** Model results for FFT effectiveness during COVID-19 pandemic.

**A: Behavioral improvement (**Δ**BHV)**					**B: Risk reduction (**Δ**RSK)**				
**Variable**	**Coeff**	**Group**			**Variable**	**Coeff**	**Group**		
		**A**	**B**	**C**			**A**	**B**	**C**
(Intercept)	β_0_	−0.441	−0.386	0.977^†^	(Intercept)	β_0_	−0.015	0.176	2.040^***^
		(0.447)	(0.550)	(0.530)			(0.518)	(0.492)	(0.501)
AGE	β_1_	0.143^*^	0.112	−0.071	AGE	β_1_	0.166^*^	0.158^*^	−0.077
		(0.070)	(0.070)	(0.072)			(0.070)	(0.062)	(0.063)
FEMALE	β_2_	0.017	−0.138^†^	−0.063	FEMALE	β_2_	0.051	−0.187^*^	−0.001
		(0.079)	(0.072)	(0.065)			(0.077)	(0.077)	(0.066)
IMMI1	β_3_	−0.051	0.111	0.079	IMMI1	β_3_	0.087	−0.024	0.092
		(0.073)	(0.071)	(0.062)			(0.097)	(0.071)	(0.063)
IMMI2	β_4_	−0.059	−0.020	0.018	IMMI2	β_4_	0.041	−0.018	−0.148^**^
		(0.062)	(0.072)	(0.056)			(0.084)	(0.070)	(0.044)
PRIOR	β_5_	0.100	0.143^†^	0.089	PRIOR	β_5_	0.125	0.123	0.178^*^
		(0.085)	(0.085)	(0.059)			(0.096)	(0.078)	(0.075)
INSTI	β_6_	0.245^*^	0.365^***^	0.154^*^	INSTI	β_6_	0.087	0.081	−0.049
		(0.101)	(0.074)	(0.077)			(0.087)	(0.074)	(0.080)
FOSTER	β_7_	0.080	0.001	−0.089	FOSTER	β_7_	−0.158^**^	−0.014	−0.164^**^
		(0.088)	(0.059)	(0.057)			(0.057)	(0.050)	(0.058)
PSYC	β_8_	0.070	0.104	0.170^**^	PSYC	β_8_	−0.031	0.163^*^	0.060
		(0.083)	(0.075)	(0.063)			(0.083)	(0.078)	(0.066)
*R* ^2^		0.135^†^	0.238^***^	0.081^*^	*R* ^2^		0.076^†^	0.110^*^	0.090^**^
		(0.070)	(0.061)	(0.032)			(0.045)	(0.048)	(0.034)

These tables summarize the standardized coefficients of the regression models used to measure FFT effectiveness during the COVID−19 pandemic. Parameters associated with behavioral improvement (ΔBHV) are presented in **(A)** whereas those associated with risk reduction (ΔRSK) are in **(B)**. Point estimates are accompanied by their standard errors below in parentheses. All estimates are pooled over 10 multiply imputed datasets using Rubin's Rule.

† *p* < 0.10;

* *p* < 0.05;

** *p* < 0.01;

*** *p* < 0.001.

Parameter estimates further revealed that clients' demographic attributes, living arrangements, and mental conditions all carried certain explanatory power in predicting treatment effectiveness. At discharge, older clients tended to have larger improvements in both behavioral ($\beta_{1}^{\text{A}}$ = 0.143, *p* = 0.040; $\beta_{1}^{\text{B}}$ = n.s.) and risk indicators ($\beta_{1}^{\text{A}}$ = 0.166, *p* = 0.018; $\beta_{1}^{\text{B}}$ = 0.158, *p* = 0.011). However, this age effect ceased to exist with the arrival of COVID-19. The results did not provide us with any clear answers regarding the significance of gender for treatment outcome. However, it did identify second-generation migrants as having made smaller progress in risk reduction after onset of COVID-19 arrived ($\beta_{4}^{\text{C}}=$ −0.148, *p* = 0.001).

Clients living in foster care tended to make smaller progress in risk reductions ($\beta_{7}^{\text{A}/\text{B}/\text{C}}$ = −0.158/n.s./−0.164, *p*^A/B/C^ = 0.006/0.777/0.005) but had comparable behavioral improvement. The reverse was true for participants who once lived in institutional care facilities, who reported larger jumps in behavioral indicators [$\beta_{6}^{\text{A}/\text{B}/\text{C}}$ = 0.245/0.365/0.154, *p*^A/B/C^ = 0.015/0.000/0.045] but had comparable risk reductions. Since the behavioral improvement scale suffered from a possible ceiling effect, individuals reporting significant gains in ΔBHV might need to have lower scores (indicating greater engagement with serious behavior problems) on the ultimate behavioral goals at the start of FFT treatment. The results suggest that FFT can achieve the ultimate goals when transitioning youths from institutional placements.

After the onset of COVID-19, clients who had received FFT or similar treatments reported larger improvements in risk indicators ($\beta_{5}^{\text{C}}$ = 0.178, *p* = 0.019), whereas those needing extra mental health services reported greater behavioral improvement ($\beta_{8}^{\text{C}}$ = 0.170, *p* = 0.007) post-COVID-19, possibly due to lower BHV scores at admission. The group differences observed above shall be interpreted conservatively, as indicated by the *post-hoc* analyses presented in Table 5.

**Table 5:** *Post-hoc* analyses for group differences.

**A: Behavioral improvement (**Δ**BHV)**							**B: Risk reduction (**Δ**RSK)**						
**Parameter**	**Group A vs B**		**Group A vs C**		**Group B vs C**		**Parameter**	**Group A vs B**		**Group A vs C**		**Group B vs C**	
	**Est**	**SE**	**Est**	**SE**	**Est**	**SE**		**Est**	**SE**	**Est**	**SE**	**Est**	**SE**
Model	−0.148	0.319	0.389	0.311	0.537	0.286	Model	0.324	1.450	2.032	1.410	1.708	1.290
(Intercept)	0.105	0.193	0.174	0.191	0.070	0.188	(Intercept)	−0.038	0.874	0.348	0.867	0.386	0.853
AGE	0.011	0.054	0.102	0.051	0.091	0.051	AGE	0.053	0.244^†^	0.542	0.231^†^	0.489	0.231
FEMALE	0.310	0.212	0.143	0.196	−0.167	0.193	FEMALE	2.022	0.959^†^	0.501	0.888	−1.521	0.876
IMMI1	−0.518	0.349	−0.377	0.319	0.141	0.313	IMMI1	1.703	1.580	0.374	1.450	−1.329	1.420
IMMI2	−0.152	0.331	−0.257	0.334	−0.105	0.283	IMMI2	0.900	1.500	2.400	1.510	1.500	1.280
PRIOR	−0.132	0.343	0.079	0.319	0.211	0.304	PRIOR	0.344	1.550	−0.059	1.440	−0.403	1.380
INSTI	−0.273	0.331	0.379	0.314	0.652	0.292^†^	INSTI	0.932	1.500	1.890	1.420	1.498	1.320
FOSTER	0.328	0.417	0.623	0.392	0.295	0.360	FOSTER	−2.984	1.890	0.986	1.780	1.998	1.630
PSYC	−0.068	0.214	−0.161	0.203	−0.093	0.197	PSYC	−1.654	0.870	−0.752	0.918	0.902	0.892

These tables present pair-wise comparisons of the marginal mean differences for parameters in the behavioral reduction model (Panel A) and in the risk reduction model (Panel B). All *p*-values are subject to Tukey adjustments.

† *p* < 0.10.

Latent growth curve models (LGM) are ideal for modeling long-term outcomes (Bollen and Curran, 2006). The intercept (*i*) in an LGM reports the starting points of one's growth trajectory, whereas its slope (*s*) indicates growth rates. While older clients in Group A experienced larger increases in behavioral indicators during the treatment phase (standardized $\beta_{1}^{\text{A}}$ = 0.143, *p* = 0.040, Table 4, Panel A), their total growth curve started at a lower point (unstandardized $\lambda_{1i}^{\text{A}}$ = −0.071, *p* = 0.006) once the maintenance phase was introduced. This unusual pattern could be the result of older clients' poor maintenance in behavioral outcomes after therapies were withdrawn. This anomaly appeared to resolve itself as Groups B and C no longer experienced behavioral regressions among older youths. A second LGM observation was related to participants from institutional care facilities. When treatment and maintenance phases were jointly considered, the Group C cohort started their recoveries at lower points ($\lambda_{6i}^{\text{C}}$ = −0.799, *p* < 0.001) but had faster growth rates ($\lambda_{6s}^{\text{C}}$ = 0.042, *p* = 0.011). Finally, the LGM intercept and slope did not correlate in any group, suggesting no “fan in” (if negative correlation between *i* and *s*) or “fan out” (if positive) among the growth curves.

Equation 1 examined FFT's ability to improve short-term behavioral outcomes at discharge, while LGMs followed clients for another 18 months to see whether such improvements were enduring. These two models jointly showed that FFT's long-term success may not be guaranteed despite apparent improvements at discharge. In this study we could also get more information on the subgroup of youth who returned from institutional care units when starting FFT. Despite lower starting points, this study highlighted the resilience of institutional youths with steeper growth trajectories—suggesting that their disadvantages were not permanent.

## Discussion

### Overview

The aim of this study was to evaluate how COVID-19 affected the target population and outcomes of FFT in Norway. Despite numerous studies demonstrating this program's efficacy before the pandemic, concerns were high over reduced success caused by the severe disruptions to social routines and service delivery formats—a hypothesis demanding urgent verification. Statistical analyses provided strong support for the stability of the target population and outcomes of FFT's even during this turbulent time. COVID-19 disrupted program delivery, but not its goals of bringing about behavioral improvement and risk reductions. Variables concerning clients' demographics, living conditions, and mental status carried certain explanatory power in predicting treatment outcomes for both immediate success and long-term maintenance.

### Responses to research questions

#### Research question 1: group differences at admissions

COVID-19 did not alter clients' characteristics when they started FFT. By dividing 518 clinical records into clients who (a) experienced no COVID-19 disruption in either treatment or maintenance, (b) experienced disruptions not in treatment but in maintenance, and (c) experienced disruptions in both treatment and maintenance, this study was able to exclude clients' initial conditions as an alternative explanation for variations in outcomes. A one-way ANOVA revealed that, at admission, neither the baseline behavioral nor risk scores differed across these three groups; variables describing individual differences such as demographic information, living arrangements, and mental conditions were also comparable at the 5% significance level regardless of COVID-19 experience.

#### Research question 2: FFT effectiveness

COVID-19 did not undermine FFT's effectiveness in Norway. Reducing youths' risk of reoffending is a key treatment goal in FFT. Using the Youth Level of Service as the risk indicator, where a high score means a higher probability of reoffending, this study found reductions in risk scores in over 90% of the clients at discharge. The arrival of COVID-19 and the imposed regime of social restrictions in Norway did not cause fluctuations in this percentage. This study also examined a related measure using the number of ultimate treatment goals (living at home, going to school/work, refraining from violence, drugs and criminal behavior) as a proxy for FFT effectiveness and found that ~50% of the clients reported positive behavioral improvements at discharge, while another 45% showed stable behavioral counts. The high proportion of clients that maintained their behavioral goals likely resulted from the limited range of the behavioral scale (possible values from 0 to 5) and a possible ceiling effect (average score of 4.1 at admission and 4.8 at discharge). The FFT service in Norway was shown to be not only effective in bringing about behavioral improvements and risk reductions but also resilient to COVID-induced disruptions.

#### Research question 3: predictors of short-term effectiveness

This study found that clients' demographic attributes, living arrangements, and mental health conditions all carried certain explanatory power in predicting FFT success at discharge. Older clients tended to have larger improvements in behavioral and risk measures—the dual indicators of treatment effectiveness—but this age effect disappeared completely after COVID-19's arrival. Although sex did not reliably predict the size of clients' improvements, COVID-19 did slow down second-generation migrants' progress in risk reduction, an observation consistent with COVID-19's differential impact on migrants (OECD, 2022).

At-risk youths may be assigned to either foster homes or institutional care facilities in Norway, and youth may receive FFT when living in a foster home or as a measure when returning home from an institution. Regression results showed that clients from foster homes tended to make smaller gains in risk reduction but comparable progress in behavioral outcomes. This suggests that youth living foster families could be in need of adapted treatments that better target their specific risk factors and socio-cultural challenges (Barkan et al., 2014; Chakawa et al., 2019). On the other hand, clients who returned home from institutional care units tended to make larger gains in behavioral measures but comparable progress in risk reduction. Due to a possible ceiling effect, clients who made large gains on the five-point behavioral scale likely started FFT with poorer behavioral conditions, a result previously reported by Robbins et al. (2016).

Lastly, clients' mental health also covaried with FFT effectiveness. Receiving mental health services was associated with higher behavioral gains—a phenomenon that emerged after onset of COVID-19. Also new to the pandemic was the favorable risk reduction profile observed in clients who returned to the program after previously receiving FFT or other treatments. The group differences should, however, be interpreted conservatively, as suggested by the *post-hoc* analyses, where the significance level was only trending (between 0.10 and 0.05).

#### Research question 4: predictors of long-term effectiveness

Variables associated with short-term treatment effectiveness were not guaranteed to remain relevant 6, 12, and 18 months later. This study showed one counterexample in Group A, where older clients' initial advantage in behavioral outcomes decayed into lower growth curves once the maintenance phase was jointly modeled.

The analysis of long-term effectiveness also shed light on participants who previously lived in institutional care facilities. Despite unfavorable prognoses declared by earlier studies (Robbins et al., 2016), COVID-19 appeared to have boosted this vulnerable cohort's recovery profile: although they started their journey behind, their trajectories were steeper than those of their peers over the long term, suggesting that— their initial disadvantages were not permanent.

Overall, the results indicates that the youth, on average, had difficulties with one of FFT's ultimate goals when they entered treatment, and that after treatment, most had achieved all the ultimate goals. This change also appears to be maintained at the 6-, 12-, and 18-month follow-ups. This applies both before and after the onset of COVID.

## Limitations and future research

Figure 2 concealed an uncomfortable truth: some clients regressed in their behavioral and/or risk measures after receiving FFT. Although this study made no provision for such a cohort, a single client left behind is one too many. Future research should focus on this vulnerable group, identifying which covariates are associated with the likelihood of treatment regression and what remedies alternates treatment, special needs education and society at large may contribute to their recovery.

A second limitation was related to the behavioral scale. At various stages of this study, the sufficiency of this five-point sum-score was questioned due to its limited range, poor discriminating power, and strong ceiling effect. Future research would greatly benefit from using a measurement device that is more sensitive to the referral problems relevant for the families receiving FFT in Norway.

## Contributions and conclusions

This study inquired into the impact of COVID-19 on FFT effectiveness in Norway. It devised a four-step analytical strategy by first ensuring equal conditions at admission, then detecting the directions and magnitudes of effectiveness indicators at discharge (Research Question 2). Next, it decomposed the treatment effectiveness into its constituents to look for factors systematically covarying with treatment success (Research Question 3). Lastly, it extended the time horizon long after discharge to expand knowledge on FFT's long-term impact on clients' social functionality (Research Question 4).

Through empirical evidence, this paper answered policymakers' concerns over a possible reduction in FFT efficacy with a conclusive “no”—the program reached its target population and remained effective throughout the COVID-19 pandemic.

## Data Availability

The data analyzed in this study is subject to the following licenses/restrictions: Due to the nature of this research, study participants did not agree for their data to be shared publicly, so supporting data is not available. However, we have included the analysis code in the Supplementary material, and all computer scripts are available on the author's GitHub website (link provided in the Supplementary material). Requests to access these datasets should be directed to DT; dagfinn.thogersen@nubu.no.
