# Supplementary material for: Functional family therapy across the COVID-19 pandemic
Source: Front Psychol. 2025 Jun 2;16:1531738. doi: 10.3389/fpsyg.2025.1531738 (PMC12171358; doi:10.3389/fpsyg.2025.1531738)
Supplement: Supplementary file 1 [file Supplementary_file_1.docx]

**Appendix A Ethics Approval**

The data material was collected by the Norwegian Center for Child Behavioral Development for quality assurance purposes and anonymized before being released to the author for analyses. Data collected before July 20, 2018, was approved by the Norwegian Data Protection Authority. After this date, the European Union’s General Data Protection Regulation came into effect, and data processing was based on informed consent. The Norwegian Regional Committee for Medical and Health Research Ethics ruled this project to be not subject to the Norwegian law regulating medical research and associated ethics requirement.

**Appendix B Additional Tables**

**Appendix C Analysis Code**

All computer scripts are made available on the author’s GitHub website: <https://github.com/torhag/Master-thesis/>.

**M*plus* Code for Behavioral Improvement (ΔBHV) as Effectiveness Measure**

**TITLE:**

Exploratory analysis of FFT effectiveness during COVID-19

Effectiveness measure chgbhv

**DATA:**

file = "FFT.dat";

ngroups = 3;

**VARIABLE:**

names =

! Var # 1--7

agecoh ageter region age ! Admin var

compstat compbin trmtleng

! Var # 8--11

immi1 immi2 female country ! Demographic

! Var # 12--20

travel venu team ! Treament var

prior insti video refer foster psyc

! Var # 21--26

innhome innvio innsch innlaw inndrug bhv0 ! Admission (T0)

! Var # 27--35

innyls1 innyls2 innyls3 innyls4 innyls5

innyls6 innyls7 innyls8 rsk0

! Var # 36--41

outhome outvio outsch outlaw outdrug bhv1 ! Discharge (T1)

! Var # 42--50

outyls1 outyls2 outyls3 outyls4 outyls5

outyls6 outyls7 outyls8 rsk1

! Var # 51—56 ! Follow-ups

home6 vio6 sch6 law6 drug6 bhv6 ! 6-month (T2)

! Var # 57--62

home12 vio12 sch12 law12 drug12 bhv12 ! 12-month (T3)

! Var # 63--68

home18 vio18 sch18 law18 drug18 bhv18 ! 18-month (T4)

! Var # 69--75

t0 t1 t2 t3 t4 chgbhv chgrsk ! Derived var

;

usevar =

chgbhv

age female immi1 immi2

prior insti foster psyc

;

missing = all (-999);

grouping = agecoh (1 = A 2 = B 3 = C);

**DATA IMPUTATION:**

impute =

chgbhv

age female immi1 immi2

prior insti foster psyc

;

ndatasets = 10;

save = fft_1_bhv_*.dat;

**ANALYSIS:**

estimator = mlr;

**MODEL:**

chgbhv on

age female immi1 immi2

prior insti foster psyc

;

**OUTPUT:**

stdyx;

**M*plus* Code for Risk Reduction (ΔRSK) as Effectiveness Measure**

**TITLE:**

Exploratory analysis of FFT effectiveness during COVID-19

Effectiveness measure chgrsk

**DATA:**

file = "FFT.dat";

ngroups = 3;

**VARIABLE:**

names =

! Var # 1--7

agecoh ageter region age ! Admin var

compstat compbin trmtleng

! Var # 8--11

immi1 immi2 female country ! Demographic

! Var # 12--20

travel venu team ! Treament var

prior insti video refer foster psyc

! Var # 21--26

innhome innvio innsch innlaw inndrug bhv0 ! Admission (T0)

! Var # 27--35

innyls1 innyls2 innyls3 innyls4 innyls5

innyls6 innyls7 innyls8 rsk0

! Var # 36--41

outhome outvio outsch outlaw outdrug bhv1 ! Discharge (T1)

! Var # 42--50

outyls1 outyls2 outyls3 outyls4 outyls5

outyls6 outyls7 outyls8 rsk1

! Var # 51—56 ! Follow-ups

home6 vio6 sch6 law6 drug6 bhv6 ! 6-month (T2)

! Var # 57--62

home12 vio12 sch12 law12 drug12 bhv12 ! 12-month (T3)

! Var # 63--68

home18 vio18 sch18 law18 drug18 bhv18 ! 18-month (T4)

! Var # 69--75

t0 t1 t2 t3 t4 chgbhv chgrsk ! Derived var

;

usevar =

chgrsk

age female immi1 immi2

prior insti foster psyc

;

missing = all (-999);

grouping = agecoh (1 = A 2 = B 3 = C);

**DATA IMPUTATION:**

impute =

chgrsk

age female immi1 immi2

prior insti foster psyc

;

ndatasets = 10;

save = fft_2_rsk_*.dat;

**ANALYSIS:**

estimator = mlr;

**MODEL:**

chgrsk on

age female immi1 immi2

prior insti foster psyc

;

**OUTPUT:**

stdyx;

**M*plus* Code for Latent Growth Model (LGM)**

**TITLE:**

LGM of FFT effectiveness during COVID-19

**DATA:**

file = "FFT.dat";

ngroups = 3;

**VARIABLE:**

names =

! Var # 1--7

agecoh ageter region age ! Admin var

compstat compbin trmtleng

! Var # 8--11

immi1 immi2 female country ! Demographic

! Var # 12--20

travel venu team ! Treament var

prior insti video refer foster psyc

! Var # 21--26

innhome innvio innsch innlaw inndrug bhv0 ! Admission (T0)

! Var # 27--35

innyls1 innyls2 innyls3 innyls4 innyls5

innyls6 innyls7 innyls8 rsk0

! Var # 36--41

outhome outvio outsch outlaw outdrug bhv1 ! Discharge (T1)

! Var # 42--50

outyls1 outyls2 outyls3 outyls4 outyls5

outyls6 outyls7 outyls8 rsk1

! Var # 51—56 ! Follow-ups

home6 vio6 sch6 law6 drug6 bhv6 ! 6-month (T2)

! Var # 57--62

home12 vio12 sch12 law12 drug12 bhv12 ! 12-month (T3)

! Var # 63--68

home18 vio18 sch18 law18 drug18 bhv18 ! 18-month (T4)

! Var # 69--75

t0 t1 t2 t3 t4 chgbhv chgrsk ! Derived var

;

usevar =

chgbhv

age female immi1 immi2

prior insti foster psyc

;

missing = all (-999);

grouping = agecoh (1 = A 2 = B 3 = C);

**DATA IMPUTATION:**

impute =

bhv0 bhv1 bhv6 bhv12 bhv18

age female immi1 immi2

prior insti foster psyc chgrsk

;

ndatasets = 10;

save = fft_3_lgm_*.dat;

**ANALYSIS:**

type = random;

estimator = mlr;

**MODEL:**

i s | bhv0 bhv1 bhv6 bhv12 bhv18 at t0-t4;

i s on

age female immi1 immi2

prior insti foster psyc chgrsk

! Mplus 8.9 is unable to produce standardized coefficient

! for varying time intervals (between T0 and T1 in this study).

! OUTPUT:

! stdyx;
